# Man-Midwifery

**Published:** 1847-11

**Authors:** 


*Man-Midwifery.*—A beardless itinerant lecturer, claiming to be an M. D,, has recently been discoursing at Boston, Mass., on the impropriety and immorality of the practice of midwifery by male accoucheurs. The Boston Medical and Surgical Journal intimates that this is a nominal object, the design being to pander to a morbid curiosity; and that the lectures, being confined to gentlemen, are attended chiefly by young clerks and apprentices, who go to listen to a recital of details connected with midwifery practice. It has become quite common of late, under the guise of science and philanthropy, to entertain the public with exhibitions and lectures, the least of the evils of which is, that they foster a corrupt taste, in the place of communicating useful information. In the instance referred to, we infer from the comments of our Boston contemporary, that a sojourn at Jericho would prepare the lecturer to discuss more understandingly the subject of which he professes to treat.

